# Severity-dependent differences in early management of thoracic trauma in severely injured patients - Analysis based on the TraumaRegister DGU®

**DOI:** 10.1186/s13049-017-0354-4

**Published:** 2017-02-02

**Authors:** J. Bayer, R. Lefering, S. Reinhardt, J. Kühle, N. P. Südkamp, T. Hammer, TraumaRegister DGU

**Affiliations:** 1 Department of Orthopedics and Trauma Surgery, Medical Center – Albert-Ludwigs-University of Freiburg, Faculty of Medicine, Albert-Ludwigs-University of Freiburg, Hugstetter Str. 55, 79106 Freiburg, Germany; 2 IFOM-Institute for Research in Operative Medicine, University Witten/Herdecke, Faculty of Health, Cologne, Germany; 3 Committee on Emergency Medicine, Intensive Care and Trauma Management of the German Trauma Society (Sektion NIS), Berlin, Germany

**Keywords:** Severely injured, Polytrauma, Thoracic trauma, Chest injury, Emergency management, Emergency procedures

## Abstract

**Background:**

Major trauma is associated with chest injuries in nearly 50% of multiple injuries. Thoracic trauma is a relevant source of comorbidity throughout the period of multiply-injured patient care and may require swift and well-thought-out interventions in order to avert a deleterious outcome. In this epidemiological study we seek to characterize groups of different thoracic trauma severity in severely injured patients and identify related differences in prehospital and early clinical management. This may help to anticipate necessary treatment for chest injuries.

**Methods:**

Patients documented between 2002 and 2012 in the TraumaRegister DGU®, aged ≥ 16 years, determined Injury Severity Score ≥ 16, and documentation from European trauma centers were analyzed. Isolated brain injury and severe head injury (Abbreviated Injury Scale_Head_ ≥ 4) led to patient exclusion. Patient subgroups were formed according to the Abbreviated Injury Scale_Thorax_ as Controls, AIS-2, AIS-3, AIS-4, and AIS-5/6. Demographic and clinical characteristics comparing the aforementioned groups were evaluated using descriptive statistics.

**Results:**

Twenty two thousand five hundred sixty five predominantly male (74%) patients, mean age 45.7 years (SD 19.3), suffering from blunt trauma (95%), and presenting a mean Injury Severity Score of 25.6 (SD 9.6) were analyzed. Higher thoracic injury severity was associated with more different thoracic injuries. The highest rate of prehospital intubation (58%) occurred in AIS_Thorax_-5/6. The worse the chest trauma, the more chest tubes were placed prehospitally, peaking at 22% in AIS_Thorax_-5/6. Out-of-hospital cardiopulmonary resuscitation was successfully performed in 11% in AIS_Thorax_-5/6 compared to 1%–3% in lesser thoracic trauma severity. Massive transfusion and emergency surgery was highest in AIS_Thorax_-5/6 compared to lesser thoracic injury (12% vs. 5%–7% and 17% vs. 3%–7%) and both were independently associated with thoracic injuries in patients with AIS_Thorax_ ≥ 4.

**Conclusions:**

We provide epidemiological data on trauma mechanism, concomitant injuries, frequencies of emergency interventions and outcome associated with different thoracic trauma severity. Prehospital and early clinical management is more complex when AIS_Thorax_ is ≥ 4. Severely injured patients with critical thoracic trauma are most challenging to take care of with highest rates in prehospital intubation, cardiopulmonary resuscitation, chest tube placements, blood transfusions as well as emergency surgery.

## Background

The percentage of patients with major trauma suffering from associated chest injuries is nearly 50% [1]. Multiple-trauma is often associated with traumatic lung injury of different severity [2], where the reported mortality of chest trauma can be as high as 60% [3]. Severe blunt thoracic trauma leads to contusions or lacerations of lung tissue, intrapulmonary bleeding, and alveolar collapse which impair pulmonary function. Additionally, respiratory function is compromised by increasing respiratory labor caused by reduced pulmonary compliance or thoracic wall instability and the impaired perfusion/ventilation relationship [4–6]. In the end, this complex pathophysiology can result in hypoxia, often necessitating endotracheal intubation [7]. A recent chest wall trauma scoring system sought to predict which patients are more likely to require mechanical ventilation and require prolonged care as well as those with a higher mortality risk [7]. However, this scoring system collects no information on concomitant injuries. Besides pulmonary failure as a major contributor to morbidity and mortality in trauma patients [8], brain death and hemorrhagic shock were major causes of early deaths in injured patients sustaining rib fractures [9]. A serious consequence of chest trauma is a pneumothorax, that can quickly become life-threatening e.g., when tension pneumothorax develops, and require immediate treatment. The published rate of pneumothorax in major trauma patients is 20.6%, and 15% of those patients experienced chest decompression [10].

In summary, chest trauma itself is the cause of considerable morbidity and may require swift and well-thought-out interventions in order to avert a deleterious outcome [11]. Of the deaths of severely injured patients, 20%–25% are attributed to chest injury [12, 13]. Thus, the combination of thoracic trauma with other serious injuries complicates already demanding patient care.

Those findings led us in this study to characterize severely injured patients with different thoracic trauma severity to identify thoracic trauma-related differences in preclinical and early clinical management. With this information, we aim to enrich our knowledge on what to expect when treating severely injured patients with serious thoracic trauma and to facilitate anticipating necessary treatment for chest injuries.

## Methods

### Database

The TraumaRegister DGU® of the German Trauma Society (Deutsche Gesellschaft für Unfallchirurgie, DGU) was established in 1993. The aim of this multicenter database is the anonymized and standardized documentation of severely injured patients.

Data are collected prospectively in four consecutive time phases from the site of the accident until discharge from hospital: A) pre-hospital phase, B) emergency room and initial surgery, C) intensive care unit (ICU) and D) discharge. Documentation includes detailed information on demographics, injury pattern, comorbidities, pre-and in-hospital management, course on ICU, and relevant laboratory findings including data on transfusion and each individual’s outcome. The inclusion criterion is admission to hospital via the emergency room with subsequent ICU care or reaching the hospital with vital signs and dying before ICU admission.

The infrastructure for the documentation, data management and data analysis is provided by AUC – Academy for Trauma Surgery (AUC – Akademie der Unfallchirurgie GmbH), a company affiliated with the German Trauma Society. The scientific leadership is provided by the Committee on Emergency Medicine, Intensive Care and Trauma Management (Sektion NIS) of the German Trauma Society. The participating hospitals submit their data anonymously to a central database via a web-based application. Scientific data analysis is approved according to a peer review procedure established by Sektion NIS.

The participating hospitals are primarily located in Germany (90%), but a rising number of hospitals in other countries is contributing data as well (at the moment Austria, Belgium, China, Finland, Luxemburg, Slovenia, Switzerland, The Netherlands and United Arab Emirates). Currently approx. 35,000 cases from more than 600 hospitals have been entered into the database per year.

Participation in TR-DGU is voluntary. For hospitals associated with TraumaNetzwerk DGU®, however, the entry of at least a basic dataset is obligatory for reasons of quality assurance. In this basic dataset some variables do not have to be reported (e.g., prehospital use of catecholamines, emergency or early surgery during early clinical management, chest tube placement).

### Patient selection

Patients documented between 2002 and 2012 in the TR-DGU were analyzed for eligibility. Patient selection was carried out according to the following criteria:

(1) online documentation of European trauma centers, (2) age ≥ 16 years, (3) ISS ≥ 16, (4) exclusion of isolated brain injuries, and (5) exclusion of severe head injury defined as AIS_Head_ ≥ 4. Injuries were graded according to the 2005 version of the Abbreviated Injury Scale (AIS) [14], and the Injury Severity Score (ISS) was calculated as described [15]. While the ISS is calculated from the three worst- affected body regions as the sum of squares of the respective AIS severity levels, the New ISS (NISS) is calculated similarly, but instead of indicating their location, the three worst injuries enter the equation irrespective of location [15, 16].

Patient subgroups were defined according to the chest injury severity (AIS_Thorax_). The first group consisted of patients with no relevant thoracic injuries (AIS_Thorax_ = 0 or 1), serving as a control group (“controls”). Group AIS-2 consisted of patients with AIS_Thorax_ = 2. Group AIS-3 consisted of patients with AIS_Thorax_ = 3. Group AIS-4 consisted of patients with AIS_Thorax_ = 4. Group AIS-5/6 consisted of patients with AIS_Thorax_ = 5 and 6, comprising the highest severity of chest trauma.

AIS_Thorax_ included all thoracic injuries coded as AIS = 4xxxxx.x [14]. Injuries to the thoracic spine were excluded, as these were coded with an AIS = 6xxxxx.x number.

### Statistical analysis

Demographic and clinical characteristics comparing the aforementioned groups were evaluated using descriptive statistics. Continuous variables are presented as mean with standard deviation (SD), while categorical variables are presented as number of cases with percentages. The respective statistics refer to patients with valid data sets only. Therefore, the total number of patients or characteristics may sometimes vary. Data for early surgery and chest tube placement in the trauma resuscitation room are not part of the basic data set, therefore not all hospitals provide this information and patient numbers vary.

Formal statistical testing would require an initial overall test (chi-squared, analysis of variance or Kolmogoroff-Smirnov) followed by pair-wise comparisons in case of significance. The number of pair-wise tests with five subgroups would be ten per variable. Since the number of patients in the five subgroups ranges from roughly 2000 to 8000, even minor differences would attain statistical significance. The 95% confidence interval in groups with 2000 cases (or more) would be about +/− 2% (or less) in case of categorical variables, and +/− 0.025*SD in case of continuous variables. For these reasons, we refrained from formal statistical testing, and analysis is mainly descriptive.

The influence of thoracic trauma severity on massive transfusion, cardiopulmonary resuscitation (CPR) and emergency surgery, respectively, was determined by logistic regression analysis with results reported using adjusted odds ratios (OR) and [95% confidence interval]. In this analysis, groups “AIS-2” to “AIS-5/6” were compared with “controls”, adjusted for AIS_Head_ ≥ 3, AIS_Abdomen_ ≥ 3 and AIS_Extremity_ ≥ 3.

All data were analyzed using SPSS, version 22.0 (IBM Inc., Armonk, NY, USA).

The present study is in line with the publication guidelines of the TR-DGU and registered as TR-DGU project ID 2011–015.

## Results

A total of 22,565 severely injured patients, mean age 45.7 years (SD 19.3) and presenting a mean ISS of 25.6 (SD 9.6) were identified for further analysis.

Most data included in this study come from patients recorded in German trauma centers (88%), while 5% of data sets were entered from trauma centers in Austria and The Netherlands, each.

### Demographics

The study group’s basic characteristics are summarized in Table 1 and consist predominantly of male (74%) patients suffering from blunt trauma (95%).

**Table 1:** Basic characteristics: Groups according to the AIS_Thorax_ (0 + 1, 2, 3, 4 and 5 + 6)

	Controls	AIS-2	AIS-3	AIS-4	AIS-5/6
	*n* = 4870	*n* = 1973	*n* = 8052	*n* = 5433	*n* = 2237
Age (years) Mean ± SD	44.2 ± 19.6	41.7 ± 18.6	45.3 ± 19.2	47.4 ± 19.0	49.3 ± 19.1
ISS Mean ± SD	21.5 ± 6.4	21.4 ± 5.9	23.5 ± 6.9	28.1 ± 9.3	39.4 ± 12.5
New ISS Mean ± SD	25.5 ± 8.3	23.5 ± 7.4	26.2 ± 7.4	33.7 ± 9.7	48.4 ± 12.6
Males	3439	1414	6009	4074	1667
	71%	72%	75%	76%	75%
Blunt trauma	4405	1855	7518	5028	1979
	94%	97%	97%	96%	92%
Penetrating trauma	296	57	270	228	173
	6%	3%	4%	4%	8%

Total numbers and percentages for gender and mechanism of injury

Total numbers in gender and trauma mechanism differ from the initial group

Regarding the injury mechanism: the majority of patients sustained their injuries during road traffic accidents, mainly in cars (*n* = 7299; 34%). All accident causes are summarized in Table 2.

**Table 2:** Mechanism of injury: subgroups according to severity of thoracic trauma

	Controls	AIS-2	AIS-3	AIS-4	AIS-5/6
	*n* = 4613	*n* = 1904	*n* = 7744	*n* = 5225	*n* = 2118
TA - Car	1045	735	2906	1847	766
	23%	39%	38%	35%	36%
TA - Motorcycle	841	338	1424	951	350
	18%	18%	18%	18%	17%
TA - Bicycle	318	99	428	305	111
	7%	5%	6%	6%	5%
TA - Pedestrian	449	141	457	319	134
	10%	7%	6%	6%	6%
Fall > 3 m	1012	384	1521	973	385
	22%	20%	20%	19%	18%
Fall < 3 m	404	108	535	360	109
	9%	6%	7%	7%	5%
Others	544	99	473	470	263
	12%	5%	6%	9%	12%

Total number and percentage of each group are given

*TA* traffic accident

After on-scene patient care by professional emergency medical service personnel (EMS) including an emergency physician, patients were admitted to a Level 1 trauma center in 70%–75% of cases, depending on the severity of sustained chest injuries. Most of the remaining patients were taken to a Level 2 trauma center (22%–26%).

### Injuries

When counting the thoracic injuries of individual patients, we observed a consistent increase in number of diagnoses in patients with higher thoracic injury severity (Table 3). Overall, 21% of patients with thoracic trauma were coded as having three or more different thoracic injuries. One patient was even documented having as many as ten different thoracic injuries (results do not include injuries to the thoracic spine).

**Table 3:** Numbers of different thoracic injuries: subgroups according to severity of thoracic trauma

	AIS-2	AIS-3	AIS-4	AIS-5/6
	*n* = 1973	*n* = 8052	*n* = 5433	*n* = 2237
Single	1637	4229	2014	518
	83%	53%	37%	23%
Multiple	336	3823	3419	1719
	17%	48%	63%	77%
Mean number of thoracic diagnoses	1.20 ± 0.47	1.66 ± 0.81	1.99 ± 0.98	2.43 ± 1.18

Total number of patients with one (single) or more (multiple) thoracic injuries and percentage within the group given as well as mean number of diagnoses ± SD per group

Since many patients suffered from multiple-injuries, we can describe relevant concomitant injuries besides their chest trauma. We defined a relevant injury as a serious (AIS ≥ 3) injury to the respective body region. The numbers and percentages of injuries to different body regions are listed in Table 4 according to the thoracic injury severity: About 22%–29% of patients with an AIS_Thorax_ ≥ 2 suffered from additional serious abdominal injury (AIS_Abdomen_ ≥ 3) and more than a third suffered serious injuries to their extremities (AIS_Extremities_ ≥ 3). Since we excluded patients with AIS_Head_ ≥ 4 in our study, serious injury (AIS_Head_ = 3) to the head occurred in 20%–33% of patients with thoracic trauma. While many patients suffered from serious injuries to multiple body regions, solely thoracic trauma occurred in between 20% (AIS_Thorax_ = 3) and more than 40% (AIS_Thorax_ ≥ 4).

**Table 4:** Concomitant injuries: subgroups according to severity of thoracic trauma

	Controls	AIS-2	AIS-3	AIS-4	AIS-5/6
	*n* = 4870	*n* = 1973	*n* = 8052	*n* = 5433	*n* = 2237
Relevant head	1485	661	2161	980	445
	31%	34%	27%	18%	20%
Relevant abdomen	1592	577	2031	1244	546
	33%	29%	25%	23%	24%
Relevant extremities	3086	1106	3705	1903	794
	63%	56%	46%	35%	36%
Isolated relevant thorax	-	-	1591	2344	931
			20%	43%	42%
GCS ≤ 8	617	287	1185	879	594
	14%	16%	16%	17%	29%

Percentages of patients within one AIS_Thorax_ group that suffered from an additional injury to another presented body region. Combinations of body regions injured were possible (multiple injured)

Isolated thoracic injuries occurred in the given percentage within one AIS_Thorax_ group, the remaining patients suffered from at least one relevant injury to other body regions

Relevant injury is AIS ≥ 3

Total number of patients with a documented GCS score differs from the initial study group

*GCS* Glasgow Coma Scale

As a marker of global cerebral function, the prehospital GCS score was ≤ 8 in 29% of patients with an AIS_Thorax_ ≥ 5; this is markedly higher than the 14%–17% of patients with an AIS_Thorax_ ≤ 4. Again, patients with severe head trauma (AIS_Head_ ≥ 4) have already been excluded from both groups.

### Prehospital management

A multitude of procedures may become necessary during prehospital treatment (Table 5). It may be necessary to secure the airway and maintain oxygenation via endotracheal intubation or chest decompression through a tube thoracostomy in patients with thoracic trauma. Our data show that prehospital intubation was performed most frequently in severely injured patients with critical thoracic injuries (AIS_Thorax_ ≥ 5), with 58% intubated before reaching the hospital.

**Table 5:** Prehospital management: subgroups according to thoracic trauma severity

	Controls	AIS-2	AIS-3	AIS-4	AIS-5/6	All patients
Intubation	1662 (4730)	754 (1929)	3274 (7843)	2320 (5288)	1268 (2183)	9278 (21973)
	35%	39%	42%	44%	58%	42%
Chest tube	44 (3626)	49 (1439)	347 (5718)	510 (3983)	345 (1564)	1295 (16330)
	1%	3%	6%	13%	22%	8%
Systolic BP ≤ 90 mmHg	697 (4257)	278 (1761)	1163 (7138)	1064 (4790)	660 (1933)	3862 (19879)
	16%	16%	16%	22%	34%	19%
Catecholamines	220 (3626)	63 (1439)	366 (5718)	398 (3983)	320 (1564)	1367 (16330)
	6%	4%	6%	10%	20%	8%
CPR	90 (4714)	19 (1924)	119 (7802)	158* (5272)	239* (2168)	625 (21880)
	2%	1%	2%	3%*	11%*	3%

Total numbers and percentages of each group are given with the total number of available datasets for each characteristic in parenthesis

Total patient numbers may vary for each prehospital procedure and characteristic because of incomplete data transmission or transmission of basic datasets. Basic datasets do not include information on chest tube placement or use of catecholamines

*: The presence of thoracic trauma was significantly associated with the need for CPR in the indicated AIS groups; adjusted for relevant (AIS ≥ 3) trauma to the head, abdomen and extremities

*BP* blood pressure, *CPR* cardiopulmonary resuscitation

The worse the chest trauma, the more chest tubes that were placed prehospitally, almost doubling with every increment in AIS_Thorax_ and peaking at 22% of patients undergoing tube thoracostomy with an AIS_Thorax_ ≥ 5.

Patients with an AIS_Thorax_ ≥ 4 were more often hypotensive outside the hospital than patients with less severe thoracic injuries, resulting in increased use of catecholamines (norepinephrine, epinephrine). About 22% of patients with an AIS_Thorax_ = 4 (respectively, 34% with an AIS_Thorax_ ≥ 5) had a documented systolic blood pressure (BP) of initially 90 mmHg or below.

Out-of-hospital CPR was necessary and successful in 11% of patients with an AIS_Thorax_ ≥ 5, compared to 1%–3% with an AIS_Thorax_ ≤ 4. The effect of thoracic trauma severity on the need for CPR was significant in AIS_Thorax_ ≥ 4 (groups “AIS-4” and “AIS-5/6”; adjusted OR 1.73 [1.33–2.26] and 6.87 [5.34–8.85], respectively).

### Early clinical management

Procedures performed during early clinical management and patient stabilization in the emergency room are listed in Table 6.

**Table 6:** Early clinical management: subgroups according to thoracic trauma severity

	Controls	AIS-2	AIS-3	AIS-4	AIS-5/6	All patients
Systolic BP ≤ 90 mmHg	582 (4399)	187 (1797)	865 (7291)	895 (4934)	599 (1971)	3128 (20392)
	13%	10%	12%	18%	30%	15%
PRBC	1332 (4844)	457 (1971)	1786 (8016)	1408 (5366)	760 (2172)	5743 (22369)
	27%	23%	22%	26%	35%	26%
Massive transfusion	353 (4844)	105 (1971)	384 (8016)	380* (5366)	264* (2172)	1486 (22369)
	7%	5%	5%	7%*	12%*	7%
Emergency surgery	213 (3277)	43 (1303)	243 (5170)	255* (3592)	230* (1337)	984 (14687)
	6%	3%	5%	7%*	17%*	7%
Early surgery	1796 (3295)	696 (1299)	2444 (5211)	1396 (3579)	473 (1336)	6805 (14729)
	55%	54%	47%	39%	35%	46%
WB-MSCT	2898 (4795)	1443 (1958)	5868 (7956)	3737 (5365)	1479 (2189)	15425 (22263)
	60%	74%%	74%	70%	68%	69%
Chest tube	84 (3581)	147 (1414)	1338 (5634)	1814 (3945)	958 (1545)	4341 (16119)
	2%	10%	24%	46%	62%	27%

Total numbers and percentages of each group are given with the total number of available datasets for each characteristic in parenthesis

Total patient numbers may vary for each procedure and characteristic because of incomplete data transmission or transmission of basic datasets. Basic datasets do not include information on emergency/early surgery and chest tube placement

Massive transfusion: ≥ 10 units of packed red blood cells

Emergency surgery: immediate surgery requiring cessation of the implemented trauma resuscitation algorithm

Early surgery: surgery performed prior to ICU admission

*: The presence of thoracic trauma was significantly associated with the need for massive transfusion or emergency surgery in the indicated AIS groups; adjusted for relevant (AIS ≥ 3) trauma to the head, abdomen and extremities

*BP* blood pressure, *PRBC* packed red blood cells, *WB*-*MSCT* whole-body multi-slice computed tomography

At presentation in the trauma resuscitation room, a systolic BP below 90 mmHg was recorded most frequently in AIS_Thorax_ ≥ 4 (18% AIS_Thorax_ = 4 and 30% AIS_Thorax_ ≥ 5).

Blood transfusions due to hemorrhagic shock during the initial resuscitation phase were most frequent in patients with critical/maximum thoracic trauma, but overall more patients had to be transfused than were actually in shock. Up to 7% of patients with AIS_Thorax_ ≤ 4 and 12% of those with AIS_Thorax_ ≥ 5 received more than 10 units of packed red blood cells (massive transfusion). The effect of thoracic trauma severity on massive transfusion was significant in AIS_Thorax_ ≥ 4 (groups “AIS-4” and “AIS-5/6”; adjusted OR 1.44 [1.23–1.69] and 2.65 [2.21–3.16], respectively).

Since many patients with an AIS_Thorax_ ≥ 5 presented in extremis, this resulted in the highest percentage of emergency surgeries (17%, e.g., thoracotomy, laparotomy, etc.) mandating cessation of the implemented trauma resuscitation algorithm. The effect of thoracic trauma severity on performed emergency surgeries was significant in AIS_Thorax_ ≥ 4 (groups “AIS-4” and “AIS-5/6”; adjusted OR 1.39 [1.14–1.69] and 3.93 [3.18–4.84], respectively).

Whole-body computed tomography (WB-MSCT) was usually performed for radiological work-up, but patients with thoracic trauma (AIS_Thorax_ ≥ 2) underwent comprehensive CT scanning more frequently. When looking at different diagnoses and their severity as reflected in the AIS_Thorax_, we tried to determine whether patients undergoing WB-MSCT had higher diagnosis-specific AIS scores than patients with plain radiographs or organ-selective CT. We did not observe higher AIS scores for flail chest, lung lacerations or hemothorax, but did detect more lung contusions (particularly AIS = 3; 25% vs. 14%), serial rib fractures (AIS = 3; 22% vs. 14%) and minor pneumothoraces (AIS = 2; 11% vs. 2%) in patients diagnosed by WB-MSCT. Interestingly enough, the WB-MSCTs did not lead to a higher percentage of chest tube placements (26% vs. 29%). However, a growing proportion of patients who underwent tube thoracostomy received chest tubes during the resuscitation period in conjunction with increasing thoracic trauma severity.

### Outcome

**Fig. 1:**
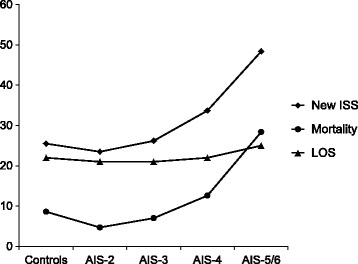
Outcome parameters depend on thoracic trauma severity. New ISS is not an outcome parameter but displayed to show the increase in overall trauma severity. New ISS is displayed in total points. Mortality is death after admission during hospital stay. Mortality is displayed in percent of patients. LOS: Length of hospital stay. Length of hospital stay is displayed in median days for surviving patients

Severely injured patients of our collective suffering from more than severe thoracic injuries (AIS_Thorax_ ≥ 4) have considerably higher scores in New ISS, as a measure of overall trauma severity, and an increase in mortality compared to minor thoracic trauma patients (Fig. 1). Patients with increased thoracic trauma severity (AIS_Thorax_ ≥ 4) tend to die earlier, while the median length of hospital stay for surviving patients (22 days vs. 25 days) is measurably longer for AIS_Thorax_ ≥ 5 only (Fig. 1).

## Discussion

We present a retrospective analysis of severely injured patients suffering from thoracic trauma of different magnitude. As in other studies [8, 10, 17–20], our population consists mainly of middle-age males with a mean ISS ≥ 16 suffering from blunt trauma. We deliberately excluded patients suffering from severe head trauma AIS_Head_ ≥ 4 to prevent confounding, since severe head trauma alone can be an indication for intubation [18]. This allowed us to distinguish between additional reasons for intubation in severely injured patients due to chest injuries.

When examining the causes that inflicted multiple injuries, we found car accidents to be particularly associated with serious thoracic trauma, in line with other reports [2, 18, 19].

It has been suggested that patients sustaining trauma to the chest and suffering from three or more rib fractures be transferred to a trauma center, as well as seriously injured patients for whom transportation to a high-volume Level 1 trauma center is recommended [9, 21, 22]. In line with the aforementioned recommendation, our patients were treated mainly at Level 1 and Level 2 trauma centers participating in the TR-DGU.

### Injuries

As one would expect in a population of severely injured patients, we detected an array of injuries to other body regions. Our moderate percentage (18%–20%) of head trauma in patients with an AIS_Thorax_ ≥ 4 is because we excluded patients with severe head trauma (AIS_Head_ ≥ 4) from our statistical assessment, since higher impact and kinematics causing major thoracic injuries will often result in significant head injuries also.

Higher percentages in concomitant abdominal and extremity injuries in severely injured patients with milder thoracic trauma are, in our opinion, attributable to the fact that to maintain an ISS ≥ 16 in the absence of significant thoracic injuries, these must have been compensated by more severe injuries to the extremities and/or the abdomen. Interestingly, about a quarter of patients with severe to critical chest injuries (AIS_Thorax_ ≥ 3) suffered from abdominal injuries that were at least serious (AIS_Abdomen_ ≥ 3) in each group.

The highest rate of patients with cerebral dysfunction we report (29%), expressed as GCS ≤ 8, occurred in severely injured patients with AIS_Thorax_ ≥ 5, compared to 14%–17% in AIS_Thorax_ ≤ 4. This is consistent with the highest rate of systolic BP < 90 mmHg in AIS_Thorax_ ≥ 5 patients, since cerebral dysfunction is an early sign of hypovolemia, in addition to the hypoxia encountered in severe thoracic trauma.

A recent study demonstrated that thoracic injuries in critical trauma patients were independently associated with a delayed diagnosis of injury. The overlooked injuries affected the chest and every other body region to varying degrees. The authors note that clinicians are easily distracted or preoccupied by more obvious or threatening conditions [23]. It is therefore not surprising that chest injuries are overlooked, even in thoracic trauma patients, as we identified higher rates of multiple injuries to the chest with higher AIS_Thorax_. Most of the patients in our investigation underwent WB-MSCT for comprehensive diagnostic workup, as recommended [24]. This is no doubt why we detected a plethora of injuries (up to 2.43 ± 1.18 injuries) in thoracic trauma by computed tomography, which is known to be more sensitive than plain radiographs [25].

### Prehospital and early clinical management

The prehospital management of severely injured patients varies with the extent of chest trauma. We noted a major increase in prehospital intubation rates in patients with AIS_Thorax_ = 4 to ≥ 5 (44% vs. 58%), consistent with previous intubation and ventilation rates reported to be between 50 and 70% depending on the severity of the initial blunt thoracic trauma [4, 26, 27].

Continuously-rising rates of chest tube insertion were documented for our patient cohorts in conjunction with increasing AIS_Thorax_. Overall, 8% (1%–22%) required preclinical chest tube placement, rising to overall 27% (2%–62%) chest tube placements during the early hospital resuscitation phase. Higher frequencies of prehospital thoracic decompression with increasing AIS_Thorax_ are in line with other reports [28]. Additionally, our rates of chest tube placements are within published rates of 44%–53% in patients presenting to the hospital with severe chest trauma [27, 29], and a rate of about 25% in patients presenting with major trauma [29, 30]. On the other hand, the rate of overall chest tube insertions in severely injured patients was recently reported to be dropping [30]. Reasons for the increase in chest tube placements we documented may be more severe pneumo- and hemothoraces with higher AIS_Thorax_ scores and, within the AIS_Thorax_ = 5 group, mandatory chest decompression because of tension pneumothorax.

When considering our patient groups’ circulatory condition, it is important to note that up to AIS_Thorax_ = 3, the reported rates of systolic BP below 90 mmHg followed by catecholamine administration did not differ. The first increase in the incidence of hypotension (systolic BP < 90 mmHg) and catecholamine use appears with AIS_Thorax_ = 4, and even more substantially in AIS_Thorax_ ≥ 5, where a third reportedly had been in shock. This might be due to the fact that, for example, relevant hemothorax involving a blood loss exceeding 1000 ml is coded as AIS_Thorax_ = 4, while tension pneumothorax is AIS_Thorax_ = 5. Injuries to the chest coded as AIS_Thorax_ < 4 seem to have an additional impact on circulation in severely injured patients.

Not contradictory is the rate of prehospital CPR in our population of severely injured patients, where about 1%–3% with an AIS_Thorax_ ≤ 4 underwent CPR. Past studies reporting on patients from the TR-DGU describe about 3% of severely injured patients undergoing CPR attempts outside the hospital [31]. There is a discrepancy in our AIS_Thorax_ ≥ 5 patients, 11% of whom underwent CPR due to greater chest trauma severity and reached the hospital showing signs of life. Furthermore, the highest rate of prehospital chest tube placement occurred in patients with AIS_Thorax_ ≥ 5, 22% of whom received this therapy. Presumably, tension pneumothoraces – one possibility for an AIS_Thorax_ = 5 score – resulted in cardiopulmonary arrest and, when properly treated via chest decompression, the appropriate management to facilitate sufficient CPR occurred as well. This might be why prehospital chest tube insertion has been observed to be a strong predictor for survival in the resuscitation of patients in traumatic cardio-respiratory arrest [32], and correlates with findings that 13% of traumatic cardiac arrests were ascribed to tension pneumothoraces [33]. Taken together, this highlights the fact that the most common management error in traumatic cardiac arrest is failing to decompress the tension pneumothorax [32, 34]. With the results of our study we can emphasize that severe thoracic trauma is a significant cause for CPR in the severely injured patient.

The application of packed red blood cells and massive transfusion was most frequent (35% and 12%, respectively) in patients with AIS_Thorax_ ≥ 5, which is consistent with the highest rate of systolic BP < 90 mmHg during the early clinical resuscitation phase. Overall, we report rates of 26% of patients requiring transfusions and 7% undergoing massive transfusion, resembling other reported rates of 24% and 5.6% in multiple-injury patients [20]. Consistent with this, we found 17% of severely injured patients with an AIS_Thorax_ ≥ 5 in such dire straits that they required emergency surgery. Additionally, we were able to show that massive transfusion and emergency surgery is significantly associated with severe thoracic trauma (AIS_Thorax_ ≥ 4) in severely injured patients.

### Outcome

Consistent with others, we report an increasing mortality with thoracic trauma severity. Albeit, our reported mortality is lower than previously published, which may in part be due to our deliberate exclusion of patients with severe head injury [3, 19]. Our reported median times of overall hospital stay are about comparable to the shorter of the previously-reported mean stays of 20 days – 38 days [19]. Interestingly, we found the longest inpatient periods with AIS_Thorax_ ≥ 5. This is in contrast to previous results [19], yet we included only surviving patients in our analysis. Patients with higher AIS_Thorax_ might experience an earlier death compared to others, thus statistically shortening the inpatient period, if not controlled for by exclusion.

### Limitations

This study has several limitations. One is its retrospective nature. Not all data were recorded on some procedures and characteristics, but those cases were still comprehensive. All hospitals participating in the TR-DGU submit to regular audits and sample tests are performed to ensure data quality. However, the documentation’s validity is not controlled by external monitoring as in prospective trials [35].

Additionally, patients from different European hospitals are included in this study. Although mainly Level 1 and 2 trauma centers contribute to this database, we cannot comment on locally implemented protocols or specialized training (e.g., ATLS®) for trauma care. But the vast majority of our patients received care in German hospitals where training in ATLS® courses and protocols has been established since 2003 [36]. During the years of our study this standardized training spread to participating hospitals, and presumably over time our patient cohort received similar early clinical treatment according to their injury severity. Nevertheless, contributing hospitals to the database change over time and so does the mixture of participating trauma center levels.

In our study, we excluded patients with severe head injury (AIS_Head_ ≥ 4) to minimize confounding and, as a result, our findings cannot be readily transferred to severely injured patients sustaining additional major head trauma.

## Conclusions

Severely injured patients are always challenging, even more so when they have suffered critical trauma to the chest. We have detected a growing number of diagnoses involving greater thoracic trauma severity, which results in more complex patients. We also noted that prehospital intubation and chest tube placement took place more often in patients presenting more severe thoracic trauma. While AIS_Thorax_ ≤ 3 did not lead to increased rates of prehospital systolic BP below 90 mmHg or catecholamine use, AIS_Thorax_ ≥ 4 did reveal a relevant impact on the circulatory system. This observation is supported by the fact that in all patients admitted to a hospital, an AIS_Thorax_ ≥ 5 correlated with the highest rate of prehospital cardiopulmonary resuscitation.

In the early clinical resuscitation phase requiring blood transfusions, mass transfusions and emergency surgeries, an AIS_Thorax_ ≥ 5 in severely injured patients frequently meant massive transfusions and emergency surgery during early hospital care. Not surprisingly, this is the most challenging group to take care of.

## Abbreviations

AIS: Abbreviated injury scale
BP: Blood pressure
CPR: Cardiopulmonary resuscitation
DGU: Deutsche Gesellschaft für Unfallchirurgie
EMS: Emergency medical service personnel
GCS: Glasgow coma scale
ICU: Intensive care unit
ISS: Injury severity score
LOS: Length of hospital stay
NISS: New ISS
OR: Odds ratio
PRBC: Packed red blood cells
SD: Standard deviation
TA: Traffic accident
TR-DGU: The TraumaRegister DGU®
WB-MSCT: Whole-body computed tomography
